# Equity impact of a choice reform and change in reimbursement system in primary care in Stockholm County Council

**DOI:** 10.1186/s12913-015-1105-8

**Published:** 2015-09-26

**Authors:** Janne Agerholm, Daniel Bruce, Antonio Ponce de Leon, Bo Burström

**Affiliations:** Department of Public Health Sciences, Karolinska Institutet, Tomtebodavägen 18a, 171 77 Stockholm, Sweden; Centre for Epidemiology and Community Medicine, Stockholm County Council, Stockholm, Sweden; Institute of Social Medicine, University of Rio de Janeiro State, Rio de Janeiro, Brazil

**Keywords:** Primary health care, Equity, Health care utilization, Health care reform, Area differences

## Abstract

**Background:**

In 2008 reforms were introduced in primary care in Stockholm County Council to increase patient choice. These reforms included changes to the reimbursement system from one that was primarily based on need-weighted capitation system (75 %) to a system largely based on fee-for-service (60 %) and freedom of establishment of primary care clinics. The new reimbursement system created incentives for producing many visits and additional primary care clinics were established, particularly in areas that were already well served. This study analyses if and how the choice reform and change of reimbursement system has affected equity in primary care consumption by investigating whether the increase in visits reflects levels of need and to what extent the reform have affected equity in health care between areas.

**Methods:**

Cross-sectional data from the public health survey in Stockholm County 2006 (*n* = 34,707) and 2010 (*n* = 30,767) were linked to individual register data on socio-demographic characteristics and health care utilization in 2007 and 2011. Information on self-reported health status and disability pension was used as indicators of need of health care. Negative binomial regression was used to analyse the differences in GP visits between the two years.

**Results:**

The total number of visits to GPs increased by 46 % from 2007 to 2011 and the proportion visiting a GP increased by 17 %. Both men and women reporting poor mental health and women with limiting longstanding illness and poor self-rated health had significantly smaller increase in number of visits than healthy women and men. Men with poor health status living in disadvantaged areas had a smaller increase than men with poor health status living in other areas of Stockholm County.

**Conclusions:**

The reform did not particularly benefit those with greater health care needs, and there are indications of a negative impact on equity in primary care after the introduction of the reform. There were signs of a lesser increase in total number of visits to GPs among those with poor mental health, among women with poor self-rated health and limiting longstanding illness, and among men living in disadvantaged areas.

## Background

The Swedish health care system is universal and primarily financed through general taxation to minimize financial barriers for access. Co-payments do exist for most types of health care, approximately 22€ for a visit to the general practitioner (GP) and 38€ for a visit to a specialist. The same level of co-payments apply to all adults, but when the yearly cost of outpatient health care services exceeds 119€ all patients have the right to free outpatient services for additional visits. The health care system is regulated by the Swedish Health Care Act (1982) and as equity in health and health care has high priority, both horizontal and vertical equity are emphasized in the act: “Health and medical services are aimed at assuring the entire population of good health and of care on equal terms. Care shall be provided with due respect for the equal worth of all people and the dignity of the individual. Priority should be given to those who are in greatest need of health and medical care” [1]. Despite these intentions, health care services are not always made available to all social groups in an equitable way.

There is an inverse social gradient in health, both on individual level and on area level: the lower the socioeconomic group, the higher mortality and morbidity [2, 3]. Consequently, the need of health care services is higher in lower socioeconomic groups and in more disadvantaged areas as well.

Equity in health care implies that health services should match needs [4]; that populations with greater needs should have more services than those with lesser needs. How to assess and measure whether this principle of equity actually applies is not straightforward. Often the number of visits to doctors [5–7] or cost of care [8, 9] are used as outcomes to assess equity in health care. However, different quality measures including patient’s experience and assessment of quality of care or access to health care have also been used [10]. For certain diseases differences between socioeconomic groups in the attainment of target levels of certain quality indicators of care such as HbA1c in diabetes, and of target levels of blood pressure have been used to assess equity in health care [11–13]. All measures address different aspects of equity in health care, and presumably more than one measure is needed for a deeper understanding of equity in health care.

Several studies on Swedish data have demonstrated inequity in utilization of health care services despite a long tradition of universal health care coverage [5, 14–17], and higher socioeconomic groups in some cases receive more complex and more expensive treatment than do lower socioeconomic groups [9].

Although the health care system might not be thought of as a main determinant of inequalities in health, it has an important role in tackling inequalities in the consequences of ill health and could potentially promote equity in health by providing care to groups in need, and by protecting lower income groups from further impoverishment due to ill health [18].

It is particularly in the first tier of the health care system that there may be a potential for health care services to help reduce inequity in health [19]. It is therefore of interest to know whether primary care services are accessible and offered in an equitable way.

### The choice reform in primary care

In 2010 a choice reform was legislated and introduced in primary care in Sweden. The focus of the reform was on giving patients free choice of provider and freedom of establishment for providers. Many county councils also changed their reimbursement systems in primary care with the introduction of the reform. In Stockholm, where the reform was introduced already in 2008, the reimbursement system was changed from a need-based resource allocation system, based primarily on need-weighted capitation (75 %) with age and area specific socioeconomic indicators used as proxies for need; to a system based more on fee-for-service (60 %), less on capitation (40 %) and now only age-weighted capitation, letting patient choice and demand direct the resource allocation to a much higher greater degree than previously [20].

The intention of the primary care choice reform was primarily to increase access to primary care. In Stockholm the total number of visits in primary care increased from 4.8 million visits in 2007 to 5.8 million visits in 2012 [21] and the number of primary care clinics increased following the reform. New clinics were established in many areas, but according to a national report, most clinics were established in areas where the service level was already high and the needs of health care were lesser [22].

The former need-based resource allocation system benefitted primary care units operating in socioeconomically disadvantaged areas. The current reimbursement system does not take into account the fact that health is poorer and disease strikes at younger ages in more deprived areas and resources are now shifting from areas with greater health care needs to areas with smaller needs [21]. This means that primary care units in areas with a population with greater health care needs now have to produce more visits in order to maintain the same amount of resources as before the reform. This could lead to lower quality of care or to prioritizing less demanding patients in order to achieve the production needed to sustain the unit’s income.

In view of the strong emphasis on equity in the Swedish Health Care Act, it is relevant to assess how the reform and the change in reimbursement system have affected groups with different levels of need of health care. To date, no scientific studies have investigated how the reform has affected equity in health care in Stockholm County. However, there have been several reports on the effect of the reform on health care utilization where also issues about equity have been partly addressed [20–24]. One of these, a survey among doctors in charge of primary health care clinics in Stockholm County, showed that only 1 % of the responders believed that the present system favoured groups with greater need and 78 % believed that the system discriminated against these groups [23].

One explanation for these findings is in the construction of the reimbursement system, which creates incentives to produce many visits, as short visits are reimbursed the same as long visits. Hence, there is a risk that doctors might prioritise patients with less complex health problems, at the expense of patients with more complex health problems.

This study investigates if and how the choice reform and change of reimbursement system has affected equity in primary care consumption, through addressing the following research questions

- Did the visits increase more in groups with greater health care needs?
- Did the visits increase more in disadvantaged areas?

## Methods

We used cross-sectional data from the Stockholm County Council’s Public Health Survey (PHS) from 2006 and 2010, a survey sent to randomly chosen individuals in Stockholm County above 18 years of age [25]. In total 65,474 participated (34,707 in 2006 (61 %) and 30,767 in 2010 (56 %)). This study was restricted to individuals aged 25–84 years (*n* = 59,065). The lower age limit was chosen to allow the use of income and educational level as a proxy for socioeconomic position. The upper age limit was chosen because the upper age limit in the 2006 survey was 84 years of age.

We obtained register data on health care utilization in 2007 for the responders of the 2006 survey and from 2011 for the responders of the 2010 survey. The health care data was obtained from the Stockholm County Council’s administrative database for analysis and follow-up of health care utilization, which contains information on all registered outpatient and inpatient care financed by Stockholm County Council. The data are anonymized through encrypted personal identity numbers.

Data on socio-demographic background characteristics and disability pension were obtained from the Longitudinal Integration Database for Health Insurance and Labour Market Studies from Statistics Sweden. This is a collection of variables from different population registers linked individually. We used the variables: age, sex, disability pension, and educational level.

All participants were informed at baseline about the survey data being linked with register data. Ethical approval for this study was obtained from the Central Ethical Review Board in Stockholm, Sweden (Dnr.:2008/1542-32).

### Variables

#### Health care utilization

Health care utilization in primary care was measured by the number of visits to GPs and by the proportion of people having one or more visits to a GP.

### Health status measures used as indicators for need of health care

#### Self-rated health and limiting longstanding illness

In the analysis of horizontal equity in health care two health status measures from the PHS were used to control for need: Self-rated health (SRH) and Limiting longstanding illness (LLI) (18). The question on self-rated health was phrased: “How do you assess your overall health? Is it: Excellent, Good, Fair, Poor or Very poor? In the analysis this variable was dichotomized into: good health (very good and good) and less than good health (fair, poor and very poor).

Participants were asked if they had a longstanding illness, problems after an accident, a handicap or another longstanding health problem. Those responding affirmatively were asked if the problem caused any difficulties in relation to the ability to work and perform other everyday activities (yes, to a high degree; yes, to some degree; not at all). Participants responding affirmatively to both questions were categorized as having a LLI.

SRH and LLI were also used to differentiate between groups with high and low needs of health care, to assess the vertical aspect of equity in health care. The two variables were combined and individuals with LLI and less than good SRH were compared to individuals with no LLI and good SRH.

Individuals with disability pension were compared to individuals with no disability pension; these analyses were restricted to individuals aged 25–64 years.

#### General Health Questionnaire-12 (GHQ12)

To differentiate between groups with and without mental health problems we used the GHQ-12, which is a screening instrument used to detect diagnosable psychiatric disorders [26]. We used the GHQ-scoring, rating each problem as either present or absent [27] and set the threshold to 2/3, where 3 or more was coded as having mental health problems and 2 or less as having no mental health problems [27, 28].

#### Disability pension

Individuals, aged 25–64, who had obtained disability pension during the year were compared to individuals aged 25–64 who did not receive disability pension during the year of interest.

#### Disadvantaged areas

In 1998 disadvantaged residential areas with high levels of unemployment, high proportion of foreign-born residents, low level of education, in the larger Swedish cities were identified for a Metropolitan Development Initiative, a programme which increased resources from state and municipal level during the period 1998–2004 to decrease segregation and improve living conditions. In these areas health is poorer and disease strikes at younger ages [29] and could therefore be regarded as areas with greater health care needs. In this study respondents living in a disadvantaged area in Stockholm County were compared to respondents living in other areas of the county.

#### Age

The association between age and number of visits was not linear across all ages and the analysis with the total study population was adjusted for age - mean age, age^2^ and age^3^. In the age group 25–64, age had a linear association with the number of visits and the analysis of relative changes in visits among those with disability pension (aged 25–64) was therefore only adjusted for age as a continuous variable.

#### Education

Educational level was categorized into 3 different levels: Primary school (9–10 years of schooling or less), Secondary school (at least one year of secondary school) and Post-secondary school (at least one year of post-secondary education).

### Statistical methods

We calculated the mean number of visits and the proportion visiting a doctor separately for men and women in different age, education and income groups, and among groups with different health care needs. When analysing the change in proportion of individuals visiting the doctor, the statistic is a ratio of two random variables (the proportion going to the doctor 2011 and the proportion going to the doctor 2007). Such an expression does not have a closed form solution for the variance. We therefore derived an approximation for the variance based on a Taylor expansion [30].

The outcome, number of visits to GPs, is a discrete variable that has a very non-normal distribution. Among different count data regression models we chose the negative binomial regression model based on goodness of fit measures, reliable estimates, and comparisons of loglikelihoods and AIC. The negative binomial regression model was used specifically to analyse the differences in GP visits between groups over time adjusted for covariates [31]. Estimates from the regression model had to be compared in a somewhat complex setting which is illustrated below using an example.

We want to compare the difference in number of visits between disadvantaged areas and the rest of the Stockholm County between 2007 and 2011. Let *μ*_*dis*, 2007_ denote the average number of visits among individuals in disadvantaged areas in 2007. An expression for the increase in number of visits from 2007 to 2011 for disadvantaged areas vs the rest of Stockholm County is then

$$ \frac{\frac{\mu_{dis,\kern0.5em 2011}}{\mu_{rest,\kern0.5em 2011}}}{\frac{\mu_{dis,\kern0.5em 2007}}{\mu_{rest,\kern0.5em 2007}}} $$

This expression of interest is a relative comparison of the gradient ‘dis’ vs ‘rest’ 2011 with the same gradient in 2007. It can be shown that using the estimates of the negative binomial regression model the expression becomes:

$$ \frac{\frac{{\widehat{\mu}}_{dis,\kern0.5em 2011}}{{\widehat{\mu}}_{rest,\kern0.5em 2011}}}{\frac{{\widehat{\mu}}_{dis,\kern0.5em 2007}}{{\widehat{\mu}}_{rest,\kern0.5em 2007}}}={e}^{b_{dis,\kern0.1em 2011}-{b}_{rest,\kern0.1em 2011}-{b}_{dis,\kern0.1em 2007}} $$

In the above coding individuals living in the rest of Stockholm County in 2007 is the reference category.

To obtain a confidence interval for the expression of interest we first calculate a confidence interval for *b*_*dis*, 2011_ − *b*_*rest*, 2011_ − *b*_*dis*, 2007_. By taking the exp-function of this confidence interval, in analogy with logistic regression models, we then obtain the confidence interval for the expression of interest [32].

## Results

### Change in number of visits

The mean number of GP visits increased in all groups from 2007 to 2011. The mean number of visits for the 2006 study population was 1.82 and 2.66 for the study population from 2010, an increase of 0.83 visits or 46 %. The increase in number of visits was greater among men (50 %) than among women (43 %) (Table 1 and 2), but this difference was not significant.

**Table 1:** Mean number of visits, proportion having one or more visits to the GP in 2007 and in 2011 and the relative changes in 2011 compared to 2007 among women

	2007		2011		Change					
	Mean number of visits	% with 1 or more visits	Mean number of visits	% with 1 or more visits	Change in mean visits	CI 95 % (Low)	CI 95 % (High)	Change in % with one or more visits	CI 95 % (Low)	CI 95 % (High)
Women										
Total	1.99	60.84	2.84	69.90	1.43	1.38	1.48	1.15	1.13	1.16
Self rated health (SRH)										
Good SRH	1.42	55.23	2.13	65.37	1.50	1.44	1.56	1.18	1.16	1.20
Less than good SRH	3.25	73.62	4.60	81.33	1.41	1.34	1.49	1.10	1.08	1.12
Limiting longstanding illness (LLI)										
No LLI	1.49	55.73	2.21	65.76	1.49	1.43	1.55	1.18	1.16	1.20
LLI	3.23	73.90	4.57	81.62	1.41	1.33	1.50	1.10	1.08	1.13
SRH and LLI										
Good SRH and no LLI	1.31	53.37	1.98	63.81	1.51	1.44	1.57	1.20	1.17	1.22
Poor SRH and LLI	3.70	76.60	5.15	83.34	1.39	1.30	1.49	1.09	1.06	1.11
Disability pension										
No disability pension	1.90	59.89	2.73	69.27	1.44	1.38	1.49	1.16	1.14	1.17
Disability pension	3.09	73.69	4.39	80.91	1.42	1.28	1.57	1.10	1.06	1.14
GHQ-12										
No mental health problems	1.83	59.17	2.70	69.09	1.48	1.42	1.54	1.17	1.15	1.18
Mental health problems	2.45	66.03	3.10	72.18	1.26	1.17	1.35	1.09	1.06	1.12
Area										
Deprived areas in Stockholm County	2.40	66.50	3.60	71.95	1.50	1.28	1.72	1.08	1.02	1.14
The rest of Stockholm County	1.97	60.57	2.80	69.81	1.43	1.38	1.48	1.15	1.14	1.17
Age										
25–44 years	1.36	54.56	1.78	61.92	1.31	1.24	1.38	1.13	1.11	1.16
45–64 years	1.87	59.78	2.52	68.48	1.35	1.28	1.42	1.15	1.12	1.17
65–74 years	2.86	71.75	4.11	82.15	1.44	1.32	1.55	1.14	1.11	1.18
75–84 years	4.28	79.82	6.16	86.03	1.44	1.31	1.57	1.08	1.05	1.11
LLI	3.23	73.90	4.57	81.62	1.41	1.33	1.50	1.10	1.08	1.13
Education										
Primary school	2.51	65.86	3.52	75.68	1.40	1.28	1.53	1.15	1.11	1.19
Secondary school	1.93	61.28	2.84	70.83	1.48	1.40	1.56	1.16	1.13	1.18
College	1.50	55.98	2.14	65.48	1.43	1.35	1.50	1.17	1.14	1.19

**Table 2:** Mean number of visits, proportion having one or more visits to the GP in 2007 and in 2011 and the relative changes in 2011 compared to 2007 among men

	2007		2011		Change					
	Mean number of visits	% with 1 or more visits	Mean number of visits	% with 1 or more visits	Change in mean visits	CI 95 % (Low)	CI 95 % (High)	Change in % with one or more visits	CI 95 % (Low)	CI 95 % (High)
Men										
Total	1.62	50.20	2.44	59.83	1.50	1.43	1.58	1.19	1.17	1.21
Self rated health (SRH)										
Good SRH	1.16	45.36	1.80	54.90	1.55	1.46	1.63	1.21	1.18	1.24
Less than good SRH	2.80	62.62	4.32	73.82	1.54	1.42	1.66	1.18	1.15	1.21
Limiting longstanding illness (LLI)										
No LLI	1.18	45.10	1.77	54.73	1.50	1.42	1.59	1.21	1.19	1.24
LLI	2.93	65.72	4.53	75.69	1.54	1.42	1.67	1.15	1.12	1.18
SRH and LLI										
Good SRH and no LLI	1.04	43.36	1.59	52.82	1.53	1.44	1.62	1.22	1.19	1.25
Poor SRH and LLI	3.32	67.80	5.24	78.51	1.58	1.43	1.73	1.16	1.12	1.19
Disability pension										
No disability pension	1.55	49.54	2.37	59.22	1.53	1.45	1.61	1.20	1.17	1.22
Disability pension	3.13	64.25	4.18	74.86	1.33	1.10	1.57	1.17	1.10	1.23
GHQ-12										
No mental health problems	1.57	49.11	2.36	59.03	1.50	1.42	1.58	1.20	1.18	1.22
Mental health problems	1.91	55.47	2.69	62.48	1.41	1.24	1.57	1.13	1.08	1.17
Area										
Deprived areas in Stockholm County	1.83	54.90	2.53	64.84	1.38	1.13	1.63	1.18	1.10	1.26
The rest of Stockholm County	1.61	49.97	2.44	59.60	1.51	1.44	1.59	1.19	1.17	1.21
Age										
25–44 years	0.81	40.51	1.07	46.21	1.32	1.22	1.42	1.14	1.10	1.18
45–64 years	1.45	48.81	2.10	58.37	1.45	1.34	1.55	1.20	1.16	1.23
65–74 years	2.78	65.87	4.00	76.27	1.44	1.30	1.58	1.16	1.12	1.20
75–84 years	4.53	77.96	5.88	82.53	1.30	1.15	1.45	1.06	1.02	1.10
LLI	2.93	65.72	4.53	75.69	1.54	1.42	1.67	1.15	1.12	1.18
Education										
Primary school	1.84	53.26	3.52	67.68	1.91	1.59	2.01	1.27	1.22	1.32
Secondary school	1.49	49.82	2.84	59.86	1.91	1.39	1.62	1.20	1.17	1.23
College	1.21	44.90	2.14	53.71	1.76	1.36	1.60	1.20	1.16	1.23

Women with mental health problems had significantly smaller increase in the number of visits than women with no mental health problems. Otherwise there were no significant differences in the change in number of visits between groups with different health care needs and between groups in disadvantaged areas compared to the rest of Stockholm County.

### Change in the proportion of individuals visiting the doctor in each group

Overall the proportion of people making one or more visits to the doctor increased from 56 % in 2007 to 65 % in 2011, a relative increase of 1.17. Each subgroup had a significant increase in the proportion making one or more visits to the doctor. Men had a significantly greater increase than women (1.19 vs 1.15) and the oldest age group (75–84 years) had a significantly smaller increase compared to all the other age groups, among both men and women.

Groups with greater health care needs had a smaller increase from 2007 to 2011 in the proportion making one or more visits to the doctor, compared to groups with lesser health care needs.

### Changes in equity in health care

Women with poor health had a significantly lower increase in number of visits than women with good health. This was true for all health care need indicators except disability pension when controlling for age and educational level. There was the same tendency for most health care need indicators among men; however, this was only significant for men with poor mental health compared to men with no good mental health (Table 3).

**Table 3:** Relative change in relative differences in number of visits between groups with different health care needs in 2011 compared with 2007, using negative binomial regression

	Model 0					Model 1					Model 2					Model 3				
	estimate	sd	95 % CI			estimate	sd	95 % CI			estimate	sd	95 % CI			estimate	sd	95 % CI		
Women																				
Poor self-rated health (SRH) vs Good SRH	0.942	0.031	0.886	-	1.001	0.947	0.030	0.893	-	1.005	0.914	0.031	0.860	-	0.972					
Limiting Longstanding Illness (LLI) vs no LLI	0.952	0.033	0.892	-	1.015	0.941	0.032	0.884	-	1.001	0.915	0.033	0.859	-	0.976					
LLI and poor SRH vs No LLI and good SRH	0.923	0.038	0.857	-	0.994	0.919	0.037	0.855	-	0.987	0.881	0.038	0.818	-	0.949					
Poor mental health vs Good mental health	0.854	0.038	0.793	-	0.921	0.885	0.036	0.825	-	0.950	0.892	0.037	0.830	-	0.959					
Disability pension vs No disability pension	1.007	0.060	0.896	-	1.132	1.004	0.059	0.894	-	1.128	0.989	0.059	0.881	-	1.111					
Deprived areas vs rest of Stockholm County	1.053	0.074	0.911	-	1.217	0.998	0.070	0.870	-	1.145	0.925	0.073	0.801	-	1.068	0.900	0.075	0.777	-	1.042
Men																				
Poor self-rated health (SRH) vs Good SRH	0.998	0.043	0.918	-	1.086	1.015	0.040	0.938	-	1.098	0.987	0.042	0.910	-	1.072					
Limiting Longstanding Illness (LLI) vs no LLI	1.027	0.044	0.942	-	1.120	0.986	0.041	0.909	-	1.069	0.948	0.043	0.871	-	1.031					
LLI and poor SRH vs No LLI and good SRH	1.032	0.052	0.932	-	1.144	1.008	0.049	0.916	-	1.110	0.964	0.051	0.872	-	1.066					
Poor mental health vs Good mental health	0.935	0.059	0.833	-	1.050	0.886	0.054	0.797	-	0.985	0.860	0.055	0.771	-	0.958					
Disability pension vs No disability pension	0.926	0.097	0.766	-	1.120	0.925	0.094	0.769	-	1.112	0.917	0.095	0.761	-	1.104					
Deprived areas vs rest of Stockholm County	0.911	0.096	0.754	-	1.100	0.805	0.089	0.677	-	0.958	0.783	0.092	0.653	-	0.939	0.825	0.096	0.684		0.995

Model 0 = The empty model

Model 1 = Controlled for age, age^2 and age^3

Model 2 = Controlled for age, age^2, age^3 and educational level

Model 3 = Controlled for age, age^2, age^3, educational level and health status (SRH, LLI and GHQ12)

Regarding equity between areas with different socio-economic characteristics, men living in disadvantaged areas had a significantly smaller increase in number of visits compared to men living in other areas of Stockholm County (Table 3). These differences were apparent primarily among those with poor health status. When stratifying by health status area differences were significant only among individuals with poor health status. Men with poor health status in disadvantaged areas had 0.68 (95 % CI: 0.50–0.92) times lower increase in visits than men with poor health status in other areas of Stockholm County (data not shown). These area differences were not significant among women.

## Discussion

The results showed that the number of visits to GPs had increased between 2007 and 2011 in all groups regardless of health status or area of residence. This was also true for the proportion of people making one or more visits to the doctor, but while there were significant differences in the increase of visits only between women with and without mental health problems, there was a tendency for all groups with greater health care needs to have a smaller increase in the proportion of people making one or more visits to the doctor. This could be due to these groups having an already high proportion of people making one or more visits to the doctor.

The results of the negative binomial analysis of changes in equity in health care showed that especially women with poor health status, both physically and mentally, and men with poor mental health had smaller increase in number of visits than the comparison groups. This is in line with a previous report on the effect of primary care reforms in Sweden, where the authors found that groups with specific health care demanding diseases had had a smaller increase in the total number of visits to GPs [24] and indicates that the general increase in number of visits might not benefit those with greater needs to the same extent as those with lesser needs.

Also men in disadvantaged areas, where levels of unemployment is higher and the proportion of individuals with low educational level greater, had a smaller increase in number of visits than men in the rest of Stockholm County, suggesting that men in disadvantaged areas did not benefit from the reform as expected. When stratifying by health status these differences were only significant for individuals with poor health status, indicating some interaction between the effect of area and health status on the rate of change in visits to the doctor (data not shown).

This could be due to lower access to primary care in more disadvantaged areas as new primary care clinics primarily have opened in inner city and well served areas and to the fact that primary care resources have been shifting from areas with greater health care needs to areas with lesser needs [21].

### Strengths and limitations

There is a lack of scientific studies about how health care reforms and especially changes in reimbursement systems affect equity in health and health care utilization. This study contributes to bridging that knowledge gap and is, to our knowledge, the first scientific study to investigate the equity perspective of the primary care reform in Stockholm County. Effects of policy changes may be very dependent on the context in which they are implemented. Nevertheless, the conclusions of this study may be useful to policy makers when changing reimbursement systems in other contexts. As most health policy documents underline the importance of equity and providing services according to need, health care reforms should be evaluated from this aspect.

Another strength of this study was the use of individually linked survey and register data, enabling the analysis of changes in GP visits by level of need of health care, to address different aspects of equity in health care. Further, the use of register data for utilization of health care in terms of number of GP visits avoids the potential recall problems associated with using survey data on consumption of health care [33].

We also had the opportunity to include data on health care utilization from the year after the survey, which avoids the bias in distinguishing between initial health status and health outcome [19].

A problem with using only two measure points in time is that it is not possible to infer that the effect observed is only due to the reform. It could also be part of a time trend, but as the increase in visits has previously been shown to be linked to the reform [21, 24] it is plausible that most of the increase in visits and the differences between groups observed in this study is due to the introduction of the reform and the new reimbursement system.

When using survey data with a response rate of 60 % or less, the findings may not reflect the entire population surveyed. Non-responders are over-represented among socially and economically disadvantaged groups, as well as in groups with greater health care needs such as individuals on sick leave [34]. Therefore this study may not correctly represent these groups.

Another limitation is the outcome measure used. The number of visits may not be the optimal indicator when studying how changes in primary care affect equity in health care. The number of visits does not necessarily show whether a person has received the care needed - sometimes one longer visit may be more beneficial to a patient than several shorter visits. One reason for the observed increase in the number of visits could be that visits which previously were longer in time, because of the fee-for service reimbursement system have been divided into several shorter visits. This might not be an improvement for the patients, as they now need to make more visits in order to meet the same health care need, this would especially be important for individuals with more complex health issues and higher health care needs. With the available data it was, however, not possible to investigate changes in the quality of care for different groups of patients. Nevertheless, the fact that resources have been distributed away from areas with high proportion of people with low socioeconomic position to areas with high proportion of people with high socioeconomic position [21] indicates that quality of care might have deteriorated in disadvantaged areas after the reform. Therefore, changes in the number of visits might mainly reflect changes in the reimbursement system and may not fully explain the effects of the reform on equity in health care.

Other studies investigating how GPs perceive the impact of the reform and the reimbursement systems on providing care according to need suggest that the reform and the change in reimbursement systems are not seen to support the intentions of equitable care according to need (25), as stated in the Swedish Health Care Act. Further studies, using other methodology and measures, are warranted of how the reform and the changes in reimbursement systems have affected the way primary care is provided in different clinics, and on how to ensure that primary care is provided based on need.

We discuss the results under the assumption that the observed changes were an effect of the reform. However, other factors, that we have not been able to control for, may have contributed. Nevertheless, our analyses focus on changes in relative differences between subgroups and we do not have reason to believe that any such factor would have differential effect on specific subgroups to a degree that could affect changes in relative differences between subgroups.

This study was restricted to studying changes in visits to GPs, as the reform was only introduced in primary care. Some of the changes observed might be compensated by visits to other specialist doctors. Further analyses taking such factors into account are needed in order to fully understand the impact of the reform.

## Conclusion

This study found no evidence that the reform particularly benefitted those with greater health care needs. On the contrary individuals with mental health problems and women with poor health status had a significantly smaller increase in primary care visits than their respective reference group, indicating that the reform had a negative impact on equity in primary care. Also men living in more disadvantaged areas had had a lower increase in number of visits than men in more affluent areas. This could reflect the fact that resources have been shifted from areas with higher health care needs to areas with lower health care needs and should be further investigated to ensure equitable primary care services in the Stockholm County Council.

## Abbreviations

GP: General Practitioner
LLI: Limiting Longstanding Illness
PHS: Public Health Survey
SRH: Self-rated Health
